# Image-Based Evaluation of In Vivo Degradation for Shape-Memory Polymer Polyurethane Foam

**DOI:** 10.3390/polym14194122

**Published:** 2022-10-01

**Authors:** Lance M. Graul, Staci J. Horn, Landon D. Nash, Thomas B. Cheung, Fred J. Clubb, Duncan J. Maitland

**Affiliations:** 1 Department of Biomedical Engineering, Texas A&M University, College Station, TX 77843, USA; 2 Department of Veterinary Pathobiology, Texas A&M University, College Station, TX 77843, USA; 3 Shape Memory Medical Inc., Santa Clara, CA 95054, USA

**Keywords:** shape-memory polymers, polyurethane foams, in vivo degradation, histopathology, embolic devices, polymeric mass-loss estimation

## Abstract

Shape-memory polymer (SMP) polyurethane foams have been applied as embolic devices and implanted in multiple animal models. These materials are oxidatively degradable and it is critical to quantify and characterize the degradation for biocompatibility assessments. An image-based method using high-resolution and magnification scans of histology sections was used to estimate the mass loss of the peripheral and neurovascular embolization devices (PED, NED). Detailed analysis of foam microarchitecture (i.e., struts and membranes) was used to estimate total relative mass loss over time. PED foams implanted in porcine arteries showed a degradation rate of ~0.11% per day as evaluated at 30-, 60-, and 90-day explant timepoints. NED foams implanted in rabbit carotid elastase aneurysms showed a markedly faster rate of degradation at ~1.01% per day, with a clear difference in overall degradation between 30- and 90-day explants. Overall, membranes degraded faster than the struts. NEDs use more hydrophobic foam with a smaller pore size (~150–400 μm) compared to PED foams (~800–1200 μm). Previous in vitro studies indicated differences in the degradation of the two polymer systems, but not to the magnitude seen in vivo. Implant location, animal species, and local tissue health are among the hypothesized reasons for different degradation rates.

## 1. Introduction

### 1.1. Measuring In Vivo Degradation of Polymeric Implants

The ability to accurately quantify and characterize the in vivo degradation of biodegradable polymeric implants is essential for comprehensive biocompatibility assessment [1,2,3]. Expected mass-loss rates, including the type of degradation products and their release rates, are identified via in vitro degradation studies. While these studies guide toxicity testing and elucidate the degradation pathways/products, they cannot be relied upon for predicting mass loss rates in vivo. Generally, in vivo studies provide more robust assessments of the implant lifetime [2,4], local and systemic toxicity of degradation products [2], and the biointerface of polymer and tissue [2,5,6,7,8,9,10,11,12,13,14,15,16,17,18,19,20,21]. Subsequent comparison of in vitro and in vivo degradation results can be used to estimate the concentration of hydrolytic and/or oxidative species at the implant site [2]. It is critical, then, that the methods used to evaluate in vivo degradation capture quantitative and qualitative data accurately and effectively.

There are several methods for measuring in vivo polymeric mass loss, all of which range in accuracy, accessibility, and applicability [3]. Formerly, we explored many of the methods (and their limitations) used for polymeric mass loss estimation, including gravimetric analysis, microcomputed tomography (μCT), magnetic resonance imaging (MRI), optical coherence tomography (OCT), scanning electron microscopy (SEM), spectroscopy, and histology [3]. While all methods listed are valid approaches, this paper will focus on the application of histology for mass-loss estimation and the relevant historical usage. Histology (histopathology) remains a key analysis for the determination of biocompatibility and is often used to evaluate tissue–implant interactions [4,9,10,11,12,13,15,16,17]. Understanding this interaction is especially important for materials that degrade oxidatively, wherein cells release reactive oxygen species (ROS) to actively (e.g., phagocytosis) or inactively (e.g., oxidative stress) degrade the implant [22]. McGough (née McEnery) et al. have extensively leveraged histology to estimate mass loss of poly(thioketal urethane) bone scaffolds due to oxidative degradation [9,10]. In these studies, the visible polymer area was quantified in the sections. These measurements were coupled with μCT volumes of the implant and tissue to quantify the tissue–polymer ratio, a critical metric of success for resorbable bone implants [9]. This trend of combinatory degradation analysis is common, as seen from Sweedy et al., where histology, SEM, and μCT were used to estimate mass loss and tissue replacement as percentages of the visible implant/tissue area [11]. Bakker et al. employed a method most similar to the one in this paper using high-magnification histology sections to calculate the percentage of the area of polymer visible across multiple timepoints [4]. The percentage of mass loss was quantified by relating the remaining area to the original implant volume [4]. All these studies evaluated porous polymer (or polymer–ceramic) implants and made volumetric assumptions concerning mass loss throughout the implant, either through μCT analyses or a priori assumptions about the original polymer implant volume. Equally important is the assumption for volumetric distribution of degradation, as this is critical to the accuracy of the methods cited and the method presented in this paper.

### 1.2. Theoretical Error of Sectional Mass-Loss Estimation Methods

Previously, we investigated the theoretical error of sectional mass loss estimation for SMP foam implants that had been randomly degraded across a wide range of possible mass-loss amounts [3]. The previous study used computational models of the PED and NED geometries and recreated the oxidative degradation morphology. The baseline error for a particular number of sections (m) was determined for each device across a range of mass-loss amounts (~3–99%). Results showed that on average, the sampling error was below 2% even with only 1–3 sections available for mass-loss estimation. This value fluctuates as the actual mass loss of the foam changes. For this study, these errors provide an additional confidence level for the estimated mass losses and will be discussed further in the context of the results. 

### 1.3. Shape-Memory Polymer (SMP) Polyurethane Foam Embolic Devices

Thermally actuated shape-memory polymer (SMP) polyurethane foams are a subset of SMPs that can transition between two geometries across a transition temperature (T_trans_), known as the shape-memory effect. For this SMP system, the foams are heated above the glass transition temperature (T_g_) and crimped to a smaller, secondary shape, and cooled below T_g_ to hold the secondary shape. They are then actuated to the primary expanded shape when heated back above T_g_. This is especially applicable for vascular applications, where porous SMP foams can be deployed endovascularly and expand to fill the vascular region once exposed to body temperature and/or blood. A SMP foam system has been developed and optimized for embolic endovascular applications [23,24]. This foam system has been evaluated through chemical characterization, mechanical testing, various biocompatibility assessments, chemical degradation analysis, in vitro cell studies, and in vivo studies [2,6,20,21,24,25,26,27,28]. Furthermore, this foam has been implemented in multiple medical device applications that are FDA-cleared or submitted for clearance. One such device is a vascular occlusion plug with a nitinol coil anchor, known as a peripheral embolization device (PED), that is intended to treat vascular insufficiency [6,26]. The second device is a foam-coated platinum coil, known as a neurovascular embolization device (NED), that is intended to treat intracranial aneurysms [20,25]. Other devices include prototypical SMP foam spheroids, also intended for treatment of aneurysms [21]. Previous in vitro chemical degradation studies and in vivo studies have identified that the SMP foams are stable under hydrolytic conditions, but oxidatively degradable [2]. These devices have been evaluated for safety via in vivo animal studies. It is helpful for future SMP foam implementation to quantify the in vivo biodegradation rate of the polymer system to guide cytotoxicity testing and approximate device lifetimes during implantation. Furthermore, this information, when paired with the histopathology assessment of the SMP foams in vivo, will provide a more complete characterization of the foam biointerface with different implant sites and species.

**Figure 1:**
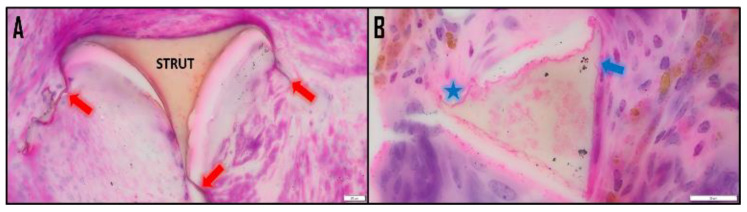
(**A**) The cross section of the foam strut is clearly visible in hematoxylin and eosin (H&E)-stained histology sections. Membranes are attached at the vertices of the strut (10 μm). (**B**) Quantifiable degradation regions are visible along the strut, including a clear scalloping pattern (star) and lone bites (arrow) (20 μm). These are sections of PED devices.

High-resolution and magnification histology sections were prepared from the explanted devices, allowing for intensive histology-driven analysis of degradation. Due to the unique biodegradation characteristics of the foam in vivo, SMP foam device mass loss can be approximated using an image-based approach with high-magnification and high-resolution histology sections. These foams are susceptible to oxidative degradation, and thus are susceptible to ROSs [2]. This activity is driven primarily by phagocytic cell types such as neutrophils, macrophages, and foreign body giant cells (FBGCs), with other cells such as myofibroblasts intensifying the oxidative environment [5,6,9,29,30,31]. As these cells interact with the foam–tissue complex, ROSs are released, ultimately resulting in degradation regions as seen in Figure 1B, similar to the historical degradation profile of implanted polyurethane foams in vivo [30,31,32]. Generally, the strut is the primary structural component and the bulk of the material mass [33,34,35]. Furthermore, all foams have a secondary structure in the pores called a membrane (see Figure 1). Membranes stretch across the faces of the pores and are connected between struts—in this arrangement, the foam is known as closed-cell [33]. When the membranes are removed or punctured, this creates an open-cell form—the most common form for medical devices. 

Through spectroscopic and SEM analysis of explanted foams, Weems et al. also identified elements of the degradation process (i.e., membrane loss and surface pitting along struts) that support the observed degradation profiles [2]. Previously, estimations of in vivo degradation from PED and NED devices were reported in two separate publications, employing the method outlined in this paper [6,20]. This method has since been refined and validated through theoretical investigations. This paper will outline the methodology developed for quantifying and analyzing in vivo degradation for SMP foams, specifically focusing on studies from embolic applications. The method itself contends with complex microstructures (i.e., struts and membranes), variable foam compositions and device geometries, and different implant locations (see Table 1). This study will determine mass-loss rates for multiple SMP foam implants and present the qualitative analysis, with comparisons to previous degradation studies and reference to previous pathology assessments [2,6,20].

## 2. Materials and Methods

### 2.1. SMP Foam Synthesis and Device Fabrication

The synthesis of SMP foams for PED devices has been detailed previously by Singhal et al. using HH30 and HH40 foam formulations [36]. In review, foams were synthesized with a three-step gas-blowing procedure. Ratios of the isocyanate prepolymer (1,6-diiscocyanatohexane is HDI (TCI America), N,N′,N′-tetrakis(2-hydroxypropyl)ethylenediamine is HPED (99% Sigma Aldrich, St. Louis, MI, USA), 2,2′,2″-nitrilotriethanol is TEA (98%, Alfa Aesar)) for HH30 and HH40 compositions, respectively, are identified in Table 2. Once cooled to room temperature, foams were mechanically reticulated according to Rodriguez et al. [23]. Foams were then cleaned and prepared for assembly according to methods outlined by Jessen et al. [6] before being assembled into final PED devices (either 6 mm or 8 mm diameter, 1 cm length). All PED devices were sterilized via electron beam radiation. The range of measured pore sizes is also listed in Table 2.

SMP foams for NED devices were crafted from foam cylinders and platinum–tungsten coils as described by Herting et al. and Boyle et al. [20,25,37]. Similar to the process for PED foams, NED foams were synthesized according to the same three-step process. However, NED foams incorporate 2,4,4-trimethyl-1,6-diiscocyanatohexane (TMHDI, TCI America, Portland, OR, USA), forming the desired TH60 composition. The ratio of HPED and TEA can be seen via Table 2. Once processed and cleaned, the foams were punched into cylinders and the coils were threaded through the center of the foams axially. Distinct from the PED devices, a neat coating of the same TH60 was applied to the foams and cured via heat. All devices were sterilized via electron beam radiation. NED devices were fabricated in various sizes, including 2, 4, and 6 mm in helical diameter and 2, 4, 6, and 10 mm in coil length [20]. The range of measured pore sizes is listed in Table 2.

SMP foams used in the porcine sidewall aneurysm study were synthesized and fabricated according to the methods outlined by Horn et al. [21]. Briefly, two foams were synthesized: foam A was synthesized from HPED, TEA, and HDI using HH60 ratios; and foam B was synthesized using HPED, TEA, HDI, and TMHDI according to the desired TM80 ratios (see Table 2). Foam B also incorporated tungsten powder (4% volume) for fluoroscopic visualization. The SMP foam composites were fabricated, wherein HH60 foams were cut into cylinders and fit into a ring of TM80 foam. Sizes of the foams were between 5.8 and 8.8 mm in diameter (outer shell) and 5.3 to 6.6 mm in height. The composites were radially conditioned via crimping at 97 °C, etched in hydrochloric acid, cleaned with 20% detergent solution, and rinsed with reverse osmosis (RO) water before being dried for 12 h at 50 °C, –76 mmHg. The range of measured/targeted pore sizes can be seen in Table 2.

### 2.2. Device/SMP Foam Implantation and Explantation

**Figure 2:**
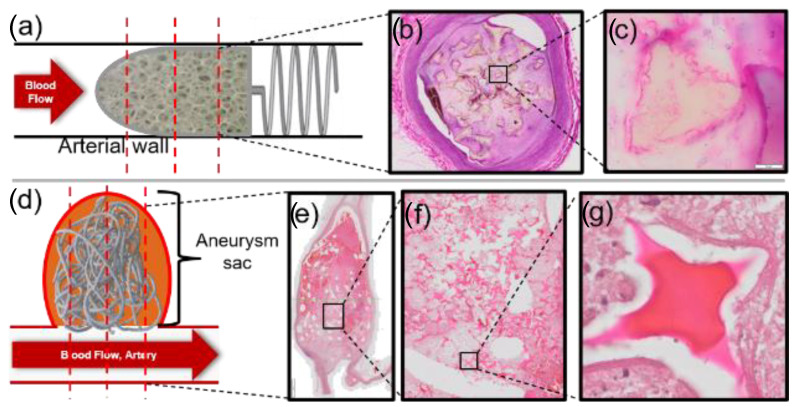
PED devices were deployed in the porcine vasculature with the foam directly in flow and the nitinol coil distal to the entry of blood (**a**). NED devices were deployed in the rabbit elastase aneurysms to fill the space—multiple devices were used of different sizing (**d**). Histological trans-sections of the explanted device(s) in tissue were scanned at high resolution and magnification (**b**,**e**). The red line denotes the general location of slices (**a**,**d**). Struts and membranes were analyzed for degradation (**c**,**f**,**g**). 2× magnification (**b**,**e**), 10× magnification (**f**), and 100× magnification (**c**,**g**) (20 μm).

The SMP foam devices were implanted according to the protocols outlined by Herting et al., Jessen et al., and Horn et al. with the NED, PED, and SMP foam composite devices, respectively [6,20,21]. In summary, PED devices were implanted in two vessels (one device per vessel) of three adult porcine specimens for 30, 60, and 90 days—9 animals in total. NED devices were implanted in rabbit elastase-induced aneurysms (various numbers of devices per aneurysm) located in the right common carotid artery. The devices were implanted for 30, 90, and 180 days, with 10, 5, and 14 animals for each timepoint, respectively. Finally, SMP foam composite devices were implanted in porcine sidewall pouch aneurysms for 90 and 180 days (2 porcine specimens per timepoint), with two foam devices per animal. For all studies, implants and the vessel (parent or occluded) were explanted and fixed in formalin for histological processing. The histological processing for each animal study is outlined in detail by Horn et al., Jessen et al., and Herting et al. (see Figure 2). Generally, the explanted tissues were fixed in formalin and prepared for paraffin embedding. Specific to NED device explants, metal coils were removed from the sections. Once sectioned and stained with hematoxylin and eosin, all slides were then scanned with an OLYMPUS Digital Microscope (Tokyo, Japan) with a 100× objective.

### 2.3. Degradation Analysis

The degradation of the polymer within each SMP foam implant was analyzed using high-resolution digital scans (see Figure 2). Whenever possible, one slide was selected from each major region of the explanted tissue (proximal, mid, distal; maximum of three slides per explant) for degradation analysis. Note that in some cases, the proximal or distal images were not feasible for evaluation due to the location of the section (e.g., section did not intersect the device). Specifically, for PED, ~3 sections were used per implant; for the SMP foam spheroid, 1 section was used per implant; and for NED, 1 section was used per implant. All sections feasible for evaluation were used for every explant. An image (or multiple images, if necessary) of the entire slide was captured for both relative membrane loss analysis and relative strut-loss analysis. For reference, all relevant terms and equations have been outlined in Table 3 and Table 4.

### 2.4. Nondegraded Foam: Sectional Features

**Figure 3:**
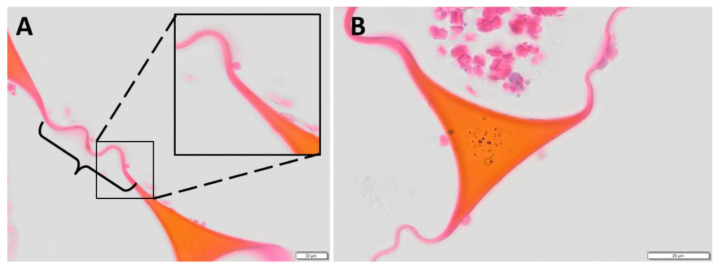
Zero-day images of SMP foam trans-sections show mostly intact membranes (**A**), bracketed region). Struts transition at vertices into membranous material, magnified in the top right of image A. Struts present with smooth edges and unperturbed surfaces (**B**). These images serve as a reference for later timepoints. Zero-day foams were stained with phosphotungstic acid hematoxylin (PTAH) (20 μm).

Nondegraded SMP foams were paraffin-embedded and sectioned, simulating the histological sectioning of devices. These sections were used as a reference for 30-day histological sections from explanted foams. As seen in Figure 3, the foam membranes that are visible have a clear connection between two strut vertices. At the juncture of strut and membrane, the material thins to 1–5 μm. Consequently, membranes are high-surface-area, low-volume components. Foam strut cross sections (Figure 3B) have a noticeable smoothness along the edges and a well-defined shape. These images aided in differentiating nondegraded struts and membranes for in vivo assessments.

Using multiple sections from 0-day samples, the visible area of membranes was compared to the visible area of struts (measurements made in FIJI (ImageJ, NIH, Bethesda, MD, USA) with the Freeform tool). In a Supplementary document, the distinction between membranes and struts is explained for the sake of quantification. From this procedure, it was estimated that membranes account for ~13% of the polymer mass, with the remaining 87% in the struts of the foam. These ratios were used for previously reported mass-loss values in Herting et al. and Jessen et al. [6,20].

### 2.5. Relative Membrane Loss Evaluation

**Figure 4:**
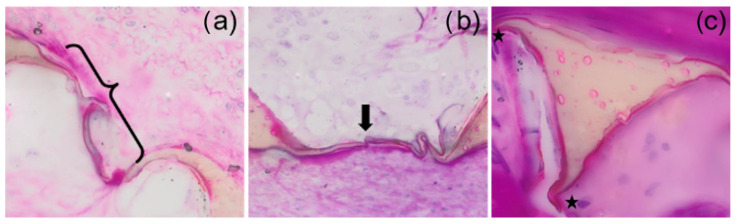
SMP foam trans-sections show the membranes at high resolution. Intact membranes ((**a**), bracket) were counted throughout the entire visible section. Membranes visible but broken ((**b**), black arrow) were counted separately to showcase the gradient of degradation. Finally, strut absent membranes at vertices ((**c**), stars) are identified as missing membranes. Tissues were stained with hematoxylin and eosin (H&E).

Membranes were divided into three groups for analysis: intact or connected membranes (compare Figure 3A to Figure 4a); broken or separated membranes; missing membranes (see Figure 4). These three groups were used to identify a relative membrane loss (see Equation (2) in Table 4) and showed the relative progression of degradation at each timepoint qualitatively. Using the overall image of the histological section, ImageJ cell counter tool was used to count the intact membranes remaining in the section, the broken/isolated membranes, and the “missing membranes” (a count based on the assumption of previous membranes at strut vertices and previous connections between adjacent struts). This evaluation was performed for all sections identified as usable for degradation analysis. An average relative membrane loss was determined for each timepoint. This evaluation is summarized by Equations (1) and (2) (see Table 4).

### 2.6. Measured Strut-Loss Evaluation

**Figure 5:**
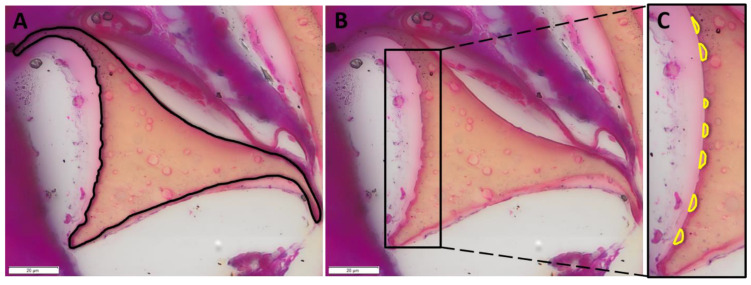
The foam struts measured for degradation were first measured for visible strut area (**A**) and then evaluated for measurable degradation area (**B**). Each degradation region was measured along the strut edge (**C**), assuming the presence of the strut edge smoothness observed from 0-day images (see Figure 3). Tissues were stained with hematoxylin and eosin (H&E) (20 μm).

**Figure 6:**
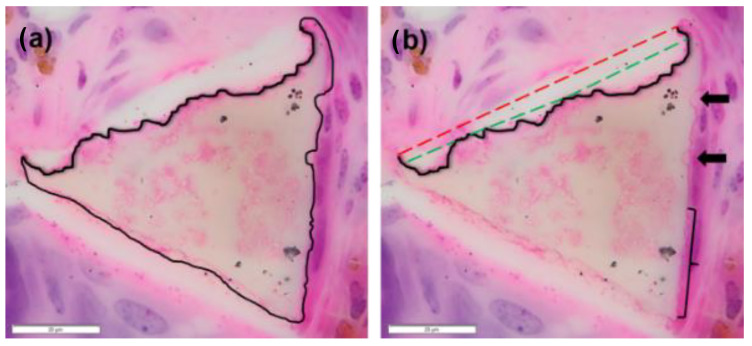
Occasional struts presented with larger degradation regions with compounded scalloping along the edges (**a**). These struts lacked a reference section on the edge. Maximal and minimal degradation lines were drafted for these cases (**b**), where the green dashed line represents the lowest possible degradation measured from strut edge assumptions, and the red dashed line represents the highest possible degradation measured. For reference, typical degradation regions are identified (black arrows) and a diminished scalloped degradation region is outlined (bracket). Tissues were stained with hematoxylin and eosin (H&E) (20 μm).

Using the Cell Counter Tool in ImageJ, the number of struts in each trans-section was counted. The average number of struts per section per timepoint was evaluated to identify potential “whole strut degradation.” If the counts were found to decrease significantly between timepoints, relative strut analysis was used to approximate whole-strut degradation (see Section 2.7 below). From the struts present in the histological section, thirty representative struts were selected to be evaluated for degradation. To evenly select throughout the section without bias to the outside or inside of the tissue site, the section was divided into a grid of nine squares. Three representative struts from each square were chosen, with an additional three selected from the top, middle, and bottom rows. These struts were then imaged in OLYVIA Virtual Microscope (OLYMPUS Corporation) at high magnification and resolution (see Figure 5A). The images were imported into ImageJ and the strut was outlined with the Freeform Tool along the visible edges of the strut (see Figure 5C). Once tracing was completed, the visible strut area was recorded. If degradation regions were visible/identified along the surface of the strut, these regions were measured by tracing around the divot or the scalloping region. This process was repeated for each of the struts from the chosen histological section. Additionally, the number of degradation regions per strut, as well as the average size of each degradation region, was tracked for all sections evaluated. This evaluation is summarized by Equation (3) (see Table 4). In rare cases, such as the foam spheroid study, struts presented with large scalloping sections and compounded degradation regions where remnant edges could not be identified (Figure 6). High and low estimations were made for these struts, as seen in Figure 6b, where a line was designated for the maximal assumed original edge and a second line demarcated the minimal assumed original edge. Referencing the 0-day image (Figure 3), struts do not present as a typical triangular cross section, rather having a curved edge from vertex to vertex. As such, the maximal assumed original edge is likely an overestimation, providing a more conservative estimation of degradation. The average between the measured degradation at the maximal and minimal was taken in these cases for use in the overall degradation calculation.

### 2.7. Relative Strut-Loss Evaluation

**Figure 7:**
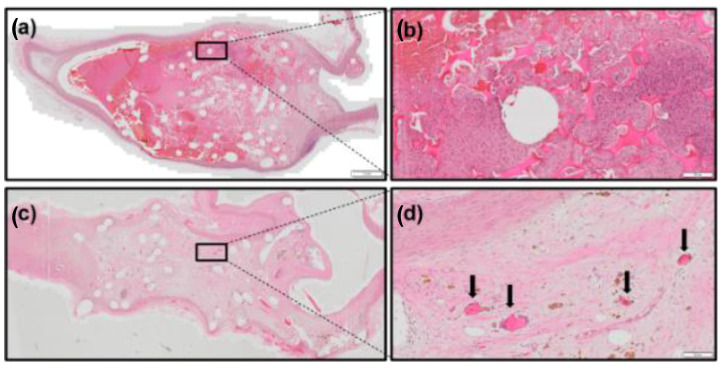
Thirty-day sections of NED devices in rabbit elastase-induced carotid aneurysms (1 μm) (**a**) present with numerous struts visible. These struts appear intact with only minor levels of surface degradation (100 μm) (**b**). Ninety-day sections show a rapid progression of degradation (**c**), with only minor groupings of struts visible, as pointed out by arrows (100 μm) (**d**). Tissues were stained with hematoxylin and eosin (H&E).

In the case of total strut loss (i.e., lower strut counts at later timepoints), an additional relative strut-loss equation was incorporated in the total degradation calculation. This evaluation procedure was developed to account for potentially large mass loss exhibited at 90 and 180 days in the NED SMP foam in the rabbit elastase-induced carotid aneurysms (see Figure 7). In this instance, the relative strut loss became the number of struts counted at each timepoint over the average number of struts observed at the 30-day timepoint. The measured strut-loss evaluation was then performed on all remaining struts (if any) to further attenuate the degradation measurement. This evaluation is summarized by Equations (4) and (5) (see Table 4). 

### 2.8. Total Degradation Calculation

The final calculation uses the membrane–strut mass ratio constants (see Table 2) as multipliers to weight the relative mass loss of each component, as seen in Equation (6) (see Table 3). For each timepoint (e.g., 30 days), the percentage of mass loss was averaged across the total available sections from multiple explants, as seen in Equation (7) (see Table 3). Note that for some explants, a large mass loss of struts was not observed, thus eliminating the need for relative strut loss (RSL).

### 2.9. Data and Statistical Analyses

When possible, all data were presented as the average ± standard deviation. For the comparison of gravimetric mass ratios with struts and membranes across the different compositions, an ANOVA was performed to identify the possibility of a significant difference. Degradation rates in vivo were reviewed for trends, with visible differences present between particular timepoints and especially between devices. Specifically, for the PED and NED in vivo studies, an ANOVA was performed within the studies between timepoints for relative membrane loss, relative strut loss, measured strut loss, and measured strut areas. Since the variances of the reported numbers were generally unequal, results were evaluated using a t-test with unequal variance (Welch’s *t*-test). A *p*-value of 0.05 was used as the standard of significance. The SMP foam spheroid studies did not have enough samples to report a standard deviation or report statistically significant results. 

## 3. Results

### 3.1. PED in Porcine Artery Model

Results from a porcine artery model with the PED device showed a steadily increasing degradation profile dominated by membrane loss (see Table 5). Membranes were typically present and intact at 30 days, while membranes at 90 days had clearly degraded. The overall strut count from each timepoint was not significantly different and total strut-degradation events were not observed in the sections (e.g., strut disintegration or debris). Generally, as the time of implantation increased, the number and size of degradation regions also increased, with more pronounced scalloping patterns at 90 days compared to 30 days. Throughout all timepoints, the presence of degradation appeared well-distributed across the section, with no regional variation. Membranes that were broken or cleaved at 30 days were typically still attached to strut vertices. By 60 and 90 days, most membranes were separated from the strut entirely and engulfed in tissue or cells. Ultimately, the 90-day timepoint served as the basis of the assumed degradation rate (worst-case scenario). This rate equivocates to a 0.11% mass loss per diem and a lifetime of approximately 3 years.

### 3.2. SMP Foam Composite Spheroid in Porcine Sidewall Pouch Aneurysm Model

Table 6 shows the measured and characterized degradation profile from a porcine sidewall model with the SMP foam spheroids. Relative membrane-loss evaluations revealed a high level of membrane degradation at 90 days. The degradation measured at 90 days was higher than that observed in the porcine artery model (~13% versus 9.42%), highlighting the importance of considering pore size, exposed surface area, foam composition, implant location, and consequent degradation rates. Additionally, the foam spheroid was a composite device, with ~150–300 μm TM80 foam on the outside portion and ~300–600 μm pore HH60 foam in the middle. Generally, the outer portions of the device showed markedly higher levels of degradation with scalloping and strut deformation. At 90 days, the strut count was consistently high across the sections evaluated. By 180 days, this number dropped significantly, especially on the outer regions of the foam device. Given the differential in strut counts across the two timepoints, this evaluation incorporated the RSL calculation to further refine the degradation rate for this polymer system. Using the 180-day data for estimation, the mass loss per day was 0.35%, approximating an in vivo foam lifetime of 285 days.

### 3.3. NED in Rabbit Elastase Aneurysm Model

The SMP foam on the NED devices implanted in the rabbit vasculature exhibited a higher degradation rate than in porcine models (see Table 7). Interestingly, there are few membranes present at 30 days and none present at 90 and 180 days. At 30 days, there are thousands of struts present within the sections (expected with small-pore, multiple-device implants) and few degradation regions visible on the struts. Struts did not exhibit signs of total strut degradation until 90 days. As such, this study required the use of the RSL to account for the large loss of struts at 90 and 180 days. At 90 days, few struts remained in the sections, typically at the neck of the aneurysm. The same observation was made at 180 days, with one case of an outlier where multiple foam strut groupings remained in regions near the aneurysm neck. The average strut count was higher at 180 days, but this has been attributed to several outliers where foam was sequestered at the neck and dome of the aneurysm, as well as a larger sample size with greater mass-loss variability. Using the 90-day data for a more conservative estimation, the devices exhibited a 1.09% mass loss per day and a foam lifetime of ~91 days.

### 3.4. Overall Analysis of Strut Areas

Given the variability of foam pore structure and the angle of sectioning throughout the foam, the average cross-sectional areas of measured struts had high standard deviations and it was difficult to quantify strut thinning, as seen in previous in vivo studies [2]. For the porcine PED studies, strut areas did not significantly differ from each other between the different timepoints. This was again true in the porcine sidewall SMP foam spheroid study, though there were clearly cases of total strut degradation—evidence that supports the use of a relative strut count. Finally, the NED devices did not present significant differences between strut areas due to the rapid degradation from 30 to 90 days. As noted in the NED data set, foams that remained at 90 and 180 days appeared to be sequestered by tissue.

## 4. Discussion

### 4.1. Limitations of the Method and Application of Error

The obvious limitation of the method stems from the lack of 0-day (pre-implantation) information. Ideally, pre-implant cross-sectional images (or total volumes) could be collected nondestructively and compared to the explanted devices. This comparison of 0-day and post-explant SMP foam cross-sectional areas would add considerable confidence in the assumptions of degradation morphology and the quantified mass loss. However, 0-day reference sections do not confer complete confidence, especially as the SMP volume is changed during implantation via tissue formation and cellular activity. This limitation is further realized when considering the variable device geometries. A potential limitation that is addressed here concerns water absorption. Water absorption is a documented and well-understood aspect of the SMP foams in this study. Yu et al. determined that the foams exhibited a maximum water uptake of 8.0% by mass after exposure to 100% relative humidity over 96 h [37]. This phenomenon was also reported by Briggs et al. as part of in vitro degradation studies [38]. However, we analyze sections of foam that have been processed for histology, including multiple dehydration washes with alcohols and xylenes. This dehydration process is assumed to remove both adsorbed and absorbed water from the foams. A third limitation concerns the variable device volumes and conformations. In the case of PED, the device geometry is relatively consistent, and sections were representative of the polymer volume. The same applies to the SMP foam spheroid studies, where the devices held a consistent spherical geometry with representative sections. However, for NED there are variable device conformations and volumes based on the implant location size and shape. A fourth limitation is the number of available sections from the explanted polymer–tissue volume. This limitation (and the third limitation) has been partially addressed via theoretical investigations of the sampling error for sectional mass-loss estimation [3]. For the PED studies, a maximum of three sections were used to estimate mass loss for one device. NED studies had one section per device for analysis. Foam spheroid studies had two to three available sections per device for analysis. Average baseline sampling errors for 1–3 available sections are still within 2%. This value is higher depending on the actual mass loss of the implant. As such, it is possible that the sampling error for the 30-day NED is closer to 5% with one section available. For PED, the error generally increases as the mass loss increases, but at 90 days (~9% mass loss) the error for estimating mass loss with three sections is nominally >0.5% [3]. Practically, these errors translate to uncertainty of implant lifetime on the order of 1–2 weeks. However, there is a further limitation to the application of error for these in vivo studies. Based on the qualitative analyses from histopathology and this study, in vivo degradation for these devices is not homogeneously distributed [6,20,21]. Regarding the spheroid implants in the porcine sidewall, degradation is higher on the outside of the implant and lower toward the center. This is due to cellular infiltration of the implant site, with higher phagocyte concentrations on the outer regions of the implant and lower concentrations internally [21]. The influence of radial bias (and other volumetric biases) in degradation on sampling error has not been explored in published research, and these biases could impact the error for these in vivo studies. A secondary concern also stems from the cell-mediated degradation sites on the foam. While macrophages are generally between 10–30 μm when activated [39,40], multi-nucleated giant cells (MNGCs) are between 40–120 μm [41] and FBGCs can be ~1 mm in diameter [42]. The larger phagocytes release more ROS and affect a larger area, thus increasing the heterogeneity of degradation in vivo. Crucially, these errors will capture the influence of heterogeneity on SMP foam mass-loss estimation accuracy.

### 4.2. Degradation Rates in Different Species and Implants: Underlying Mechanisms

The rates of degradation across the different foam implants and species are significantly different. PED in vivo foam degradation rates posit a lifetime twelve times longer than the NED implants. This is coupled with 90-day pathology assessments from Jessen et al. that state the presence of acute inflammatory cells diminished from 30 to 90 days while cells related to healing increased in number, indicative of the desired wound healing response [6]. From a toxicity standpoint, the relatively slow release of material in PED devices intimates a lower concentration of degraded polymer in local tissue than predicted in vitro. This aids in establishing the safety of the PED device. For NED, the relatively rapid degradation is intriguing, but not unexpected based on previous in vitro studies [2]. Since rigorous toxicity testing is based on accelerated rates, the rate seen in vivo falls well within an acceptable range. Moreover, pathology assessment of the explants reveals the general occlusion and subsequent healing of the aneurysm site with NED implants—results that support the biocompatibility of the observed degradation rate [20]. The studies employing the composite SMP foam spheroid produced a moderate degradation profile compared to the NED and PED. Rapid degradation of the outer material (TM80) was intriguing and suggested a positive relationship between decreasing pore size and rate of degradation. For the SMP foam spheroid studies, the outer portion of the device contained tungsten-loaded TM80 foam. Metallic additives are known to accelerate the decomposition of some polymers. Since a heightened degradation rate was observed in this foam compared to the inner core of the same device, future work should explore the in vitro degradation profile of tungsten-loaded foams and compare the results to typical foam compositions to confirm the potential catalytic effect of tungsten on SMP foam degradation. The larger pore foam (HH60) comprised the inside of the device and showed a diminished rate of degradation. It is possible that cellular infiltration of the device was limited in early stages of the implantation, yet cell counts from 90 and 180 days suggest an even distribution of macrophages, fibroblasts, and other wound-healing cell types throughout the volume of the implant [21]. It must be noted that the average mass loss at 180 days (97.3%) was lower than at 90 days (98.5%) for the NED. However, the standard deviation was much higher at 180 days (3.08%) than at 90 days (0.22%). At 90 days, only five samples were able to be evaluated for degradation, whereas fourteen samples were evaluated for 180 days. When considered in the context of visible degradation, none of the 90-day samples had 100% mass loss, whereas (3/14) had 100% mass loss by 180 days. Two outliers in the 180-day group had greater than 10% of struts remaining; these struts were isolated in the tissue and in some cases were completely undegraded. Even with the fluctuation in degradation, the healing scores were not significantly different for samples with 100% mass loss versus samples with remaining struts at 180 days. In general, tissue ingrowth was framed as percent occlusion and healing scores from pathology analysis. In the case of the PED porcine artery study, tissue fully occluded (percent occlusion of 100%) the vessel by 60 days, when only ~6.91% of the foam had degraded [6], compared to the NED rabbit elastase carotid aneurysm study, where after 90 days near-total mass loss had occurred and the degree of occlusion (and healing score) was also high [20]. For example, the relative area of collagen deposition steadily increased from 30–180 days, even as the foam completely degraded. In all three studies examined in this paper, tissue ingrowth increased as the foam degraded, but the rate of tissue ingrowth was independent of degradation rates. A future study could implant different SMP foams (e.g., different compositions, pore sizes, etc.) in the same vascular occlusion animal model to determine the relationship (if any) between tissue ingrowth and the rate of degradation. 

There are multiple hypotheses for the underlying mechanisms of variable degradation rates, especially with respect to different species. A myriad of known factors directly affects the rate of degradation, such as total available surface area. Due to the high porosity of the SMP foams, the available surface area of the foams is higher, potentially allowing for more surface-driven events (e.g., increased cellular interaction). Notably, the NED devices employ a foam with smaller pores and higher total surface area. Assuming the same volume of foam, the TH60 foams employed in the NED studies would have twice the available surface area compared to the HH30 and HH40 foams from the PED devices, based on surface area approximation equations developed by Weems et al. [27]. Pore size is another significant foam feature that naturally dictates flow of blood and fluid, but also cellular activity. For example, uniform small pores can impact macrophage infiltration as well as macrophage phenotype, potentially influencing the rate of healing and phagocytic activity [7,43]. This impact extends to fibroblasts and the ingrowth of tissue in general. The location of the implant harbors immense importance, as the devices are used for different applications. For example, PED devices are intended for use to treat vascular insufficiency, blocking blood flow permanently in damaged or diseased vasculature. The device is placed in direct flow of blood and relies on rapid clotting. With direct flow, there is the potential for a wash-out effect, especially at the entry of flow to the device. This would prevent immediate protein adsorption or cellular attachment in the initial stages of implantation, slowing down the process of inflammation and subsequent degradation. The same principle applies to the NED devices, where occasional groupings of foam struts survived at the aneurysm extremities (i.e., aneurysm dome and neck) and appeared to have negligible degradation. A recent study by Chau et al. quantified macrophage polarization and activation in explanted tissue samples from the same rabbit carotid aneurysm model used in this study. Curiously, they found that the concentration of macrophages, M1 and M2, were higher for the NEDs versus the bare metal coils (control device), though this could be attributed to a higher presence of MNGCs in vivo [20,28]. 

Chemical analyses are an equally important aspect of degradation evaluation. We have performed extensive chemical analyses for both in vitro and in vivo degradation studies of our SMP foams. Weems et al. identified spectroscopic shifts in the material that were subsequently related to the gravimetric analyses [2]. Additionally, solid-state ^13^C nuclear magnetic resonance (NMR) was used in conjunction with FTIR to confirm specific peak assignments in the spectroscopic results [2]. Briggs et al. similarly used FTIR to identify and confirm spectroscopic shifts as the result of degradation [38]. While we have used high-performance liquid chromatography (HPLC) in an unpublished work to analyze our foams, we have not done so for degrading foams. This method would be a strong addition to future evaluation of the in vivo degradation of our polymers.

An intriguing differentiator between the porcine vessel (PED) study and the porcine sidewall aneurysm (SMP spheroid) was the overall health of the local tissue. The PED devices were deployed in otherwise healthy vasculature to procure occlusion, while the porcine sidewall aneurysms were created via anastomosis of a vein pouch to the carotid artery [6,21]. Injury to the tissue could have induced an early inflammatory response, one not related to the presence of SMP foam. This is perhaps more relevant to the NED studies, where the aneurysm sac was created through the removal of inner carotid elastin and subsequent ballooning of the region [20]. This raises an important consideration when implanting devices in diseased and/or injured tissue where acute or chronic inflammation may be present, as this could accelerate cell-mediated degradation of the SMP foams.

A prominent limitation across the studies is the species of animal used in the model. Rabbit and swine models have been used extensively as aneurysm models, and there are defining features of each model that may factor into the degradation rate. Porcine sidewall aneurysms (and porcine vasculature in general) tend to heal well regardless of material, which would suggest a less realistic in vivo response compared to humans and potentially misleading degradation rates [44]. Dai et al. have shown that rabbit elastase-induced aneurysms heal more closely to human aneurysms as opposed to porcine sidewall aneurysms when treated with bare platinum coils (nonpolymeric) [45]. More specifically, when comparing in vivo responses to polyurethanes in rabbits and other species, rabbits tend to form FBGCs at a higher concentration, especially during the shift from inflammation to healing in the region [32]. Rabbits also carry an additional white blood cell class, heterophils, that are not present in humans or swine [20,45]. The increased presence of reactive oxygen species (ROS) producing cells within rabbits could intensify and accelerate the oxidative degradation of the SMP system. It is possible that ROS production inherently differs between the two species as a result of different inflammatory cell concentrations. ROS are critical components to cell signaling, especially for cells involved in acute and chronic inflammation phases [5,9,46,47,48]. Myofibroblasts, cells responsible for the production and remodeling of connective tissue matrices, generate hydrogen peroxide when crosslinking collagen via lysyl oxidase [49,50]. Reactive nitrogen species are a relevant consideration for vascular devices, as nitric oxide (NO) regulates the tone of vasculature and is regularly used in endothelial cell signaling [51]. While not considered in previous SMP foam degradation studies, the abundance of NO in vivo could play a minor role. Identifying cellular ROS production, especially H_2_O_2_, will be important for developing accurate mass loss kinetic models.

PED devices are approximated to survive for three years in vivo; however, recent in vitro studies for HH30 and HH40 foams have shown a faster degradation profile under 3% H_2_O_2_ (current ISO-10993-13 standard for emulating in vivo ROS environment) [38,52]. Conversely, NED foams approximated to last ~90 days degraded faster than TM60 foams in 3% H_2_O_2_ (~128 days to 100% mass loss). Based on studies from Weems et al., it was observed in vitro that specific foam compositions (HH60 and TH60) degraded in 3% H_2_O_2_ approximately twice as fast compared to in vivo estimates [2]. Considering the marked disparity between in vitro and in vivo rates, the assumed standard of 3% H_2_O_2_ seems inaccurate for different species and different foam compositions [53]. Examining the homogeneity of in vitro chemical degradation as opposed to heterogeneity of cell-driven in vivo degradation will be crucial to accurately assessing the mass-loss kinetics of the SMP foams. This combinatory degradation profile should also be implemented in higher-fidelity modeling of mass loss to further refine error calculations. 

Future investigation of the foam design parameters will be critical to understanding many of the proposed mechanisms. There are several parameters for our SMP foam that can be modified to influence the degradation rate overall. Many of these parameters were explored in great depth by Weems et al. The foam composition can be modified to impact the hydrophobicity/hydrophilicity of the material surface, the thermal transition temperature (T_g_), and presence/accessibility of oxidatively labile sites in the polymer network (HDI vs TMHDI-based materials) [2]. Significantly, it was found that the rate of degradation was generally dependent on T_g_, which in turn can be tuned by modifying the foam chemistry. The foam porosity impacts the degree and rate of blood/fluid diffusion throughout the volume of the foam. Related to this, through reticulation of the membranes in the pores, we can control the degree of openness in the foam, which in turn influences the flow of blood and movement of cells through the foam, creating degradation gradients [2]. The total available surface area of the foam (which is related to pore size) has a significant impact on the degradation of the foams, more so than hydrophobicity; foams with similar T_g_ but a higher surface area degraded faster in vitro, and likely would degrade faster in vivo in the same animal model [2].

## 5. Conclusions

A method for estimating and quantifying SMP foam mass loss using histological sections was developed and can be used in conjunction with histopathology analyses, affording a powerful dual examination of foam functionality in vivo. The variable rates of in vivo degradation seen in the three SMP foam devices presented in this study reveal the importance of understanding not only the kinetics of the degradation, but also underlying factors that drive the differential rates of mass loss. While the discussion has covered some of the major factors affecting the in vivo degradation profile, it is the goal of future studies to examine these (e.g., ROS, membrane/strut kinetics, pore size, and surface area) more intently to delineate true mass-loss kinetics. It is important to note that in the case of these occlusive devices, degradation is a desired feature, whereupon the removed foam is replaced with a collagenous tissue matrix during the wound-healing process. From a regulatory standpoint, the rate of exchange of tissue for foam is a strong indicator of the compatibility of the SMP devices. Coupled with the numerous previous articles asserting the biocompatibility of the SMP foams [2,6,20,21,24], this work adds quantitative and qualitative support for the functionality and safety of the SMP foam embolic devices. 

## Figures and Tables

**Table 1:** Various animal studies with implant locations as well as the SMP foam devices implanted.

Implant Location/Model	Device (Foam Type)	Details	Duration (Days)
Porcine artery [6]	PED (HH30/HH40)	One device per vessel	30, 60, 90
Rabbit elastase aneurysm [20]	NED (TH60)	Multiple devices per aneurysm	30, 90, 180
Porcine sidewall pouch [21]	Foam ball (HH60 andTM80)	One or two implants per pouch	90, 180

**Table 2:** SMP Foams with identifying chemistry, pore size, and associated device.

Foam Name	Associated Device	Pore Sizes	Chemical Composition
HH30/40 [6]	PED	[800–1500] μm	HDI; HPED 30/40%; TEA 70/60%
TH60 [20]	NED	[150–400] μm	TMHDI; HPED 60%; TEA 40%
HH60 and TM80 [21]	Foam spheroids	[400–800] μm	HDI, HPED 60%, TEA 40%
		[150–400] μm	TMHDI, HPED 80%, TEA 20%

**Table 3:** Representative mathematical terms used in Table 4 and their corresponding definitions.

Term	Definition
M	Membranes counted (e.g., missing, broken, intact)
S	Struts counted (e.g., missing, intact)
MSL	Measured strut area loss
RML	Relative membrane loss
RSL	Relative strut loss
K_S_	Assumed strut mass percent of foam
K_M_	Assumed membrane mass percent of foam
i	Number of histology sections per timepoint

**Table 4:** The equations listed below represent the degradation quantification method, inclusive of large mass-loss possibilities.

#	Equation
(1)	$M_{Total}=M_{Missing}+M_{Broken}+M_{Intact}$
(2)	$RML=\frac{M_{Missing}}{M_{Total}}*100\%$
(3)	$StrutArea_{Total}=StrutArea_{Measured}+DegArea_{Measured}$
(4)	$MSL=\frac{DegArea_{Measured}}{StrutArea_{Total}}*100\%$
(5)	$RSL=\frac{\left(S_{30day}-S_{counted}\right)}{S_{30day}}*100\%$
(6)	$\%Deg_{Total}=RML*K_{M}+\left(RSL+MSL*\left(1-RSL\right)\right)*K_{S}$
(7)	$\%Deg_{XDays}=\frac{\sum_{0}^{i}\%Deg_{Total,i}}{i}$

**Table 5:** PED degradation quantification/qualification in a porcine artery model ^1^.

Timepoint (Days)	Mass Loss (%)	RML (%)	Avg. Foam Strut Count	Qualitative Assessment
30 (n = 11)	3.22 ± 3.90	21.3 ± 7.05	62.7 ± 12.9	-Membranes mostly broken/separated and -~50% membranes curled/isolated due to cellular activity -Isolated degradation regions along strut edges
60 (n = 12)	6.91 ± 4.97	48.4 ± 8.53	63.7 ± 11.4	-Membranes are mostly degraded, with the rest broken and/or isolated due to cellular activity or tissue ingrowth -Increased number of degradation regions per strut, as well as increased number of struts with degradation regions
90 (n = 17)	9.42 ± 7.05	64.3 ± 13.9	59.1 ± 12.9	-Majority of membranes are degraded; remaining are broken or isolated -Continued increase in number of degradation regions and number of struts presenting degradation (scalloping pattern)

^1^ Adapted with permission from S.L. Jessen, M.C. Friedemann, A.M. Ginn-Hedman, L.M. Graul, S. Jokerst, C.B. Robinson, T.L. Landsman, F.J. Clubb, D.J. Maitland, Microscopic Assessment of Healing and Effectiveness of a Foam-Based Peripheral Occlusion Device, ACS Biomater. Sci. Eng. 6 (2020) 2588–2599. https://doi.org/10.1021/acsbiomaterials.9b00895 (accessed on 23 August 2022). Copyright 2019 American Chemical Society.

**Table 6:** Foam spheroid degradation quantification/qualification in a porcine sidewall pouch model.

Timepoint (Days)	Mass Loss (%)	RML (%)	Avg. Foam Strut Count	Qualitative Assessment
90 (n = 2)	12.9	86.4	410	-Near-total membrane loss at this stage; few intact membranes -Tungsten-doped struts showed higher degradation (close to walls of the explant tissue) -Higher degradation on outer zone of device
180 (n = 2)	63.3	95.3	174	-Near-total membrane loss at this stage; few intact membranes -Continued increase in number of degradation regions and number of struts presenting degradation -Total strut loss observed in over half of struts; all strut loss near the wall of pouch

**Table 7:** NED degradation quantification/qualification in a rabbit elastase aneurysm model.

Timepoint (Days)	Mass Loss (%)	RML (%)	Avg. Foam Strut Count	Qualitative Assessment
30 (n = 10)	13.6 ± 2.75	100	1830 ± 460	-Total membrane loss; very few remnants, not quantitatively significant -Degradation regions visible on many struts
90 (n = 5)	98.5 ± 0.22	100	29.4 ± 4.16	-Total membrane loss -Near total strut loss; pockets of struts remain near the neck of the aneurysm and occasionally the dome -Foam struts in parent artery still present
180 (n = 14)	97.3 ± 3.08	100	53.0 ± 51.4	-Total membrane loss -Some explants with total strut loss (100%) -Many explants have pockets of foam near the neck of the aneurysm

## Data Availability

Data may be made available upon reasonable request.

## Supplementary Materials

The following supporting information can be downloaded at: https://www.mdpi.com/article/10.3390/polym14194122/s1, Figure S1: Non-degraded membranes; Figure S2: Non-degraded strut edges; Figure S3: 0-day strut versus degraded strut.
