# Supplementary material for: Image-Based Evaluation of In Vivo Degradation for Shape-Memory Polymer Polyurethane Foam
Source: Polymers (Basel). 2022 Oct 1;14(19):4122. doi: 10.3390/polym14194122 (PMC9571375; doi:10.3390/polym14194122)
Supplement: Supplementary file 1 [file polymers-14-04122-s001.zip › polymers-1904103-supplementary.pdf]

Supplementary Document

Membrane Presence Assumption

Membranes that have not been reticulated during device manufacturing are assumed to be intact prior to implantation. This is based on 0-day sections from representative SMP foams

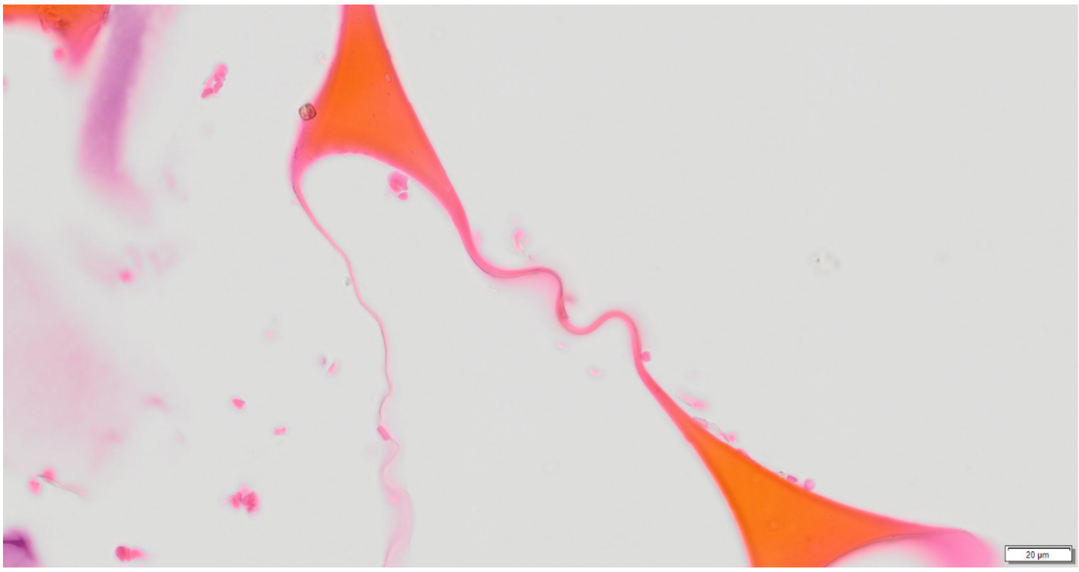

Figure S1: Membranes are typically found connected between two strut vertices, when viewed from a two-dimensional perspective in foams that have not been implanted or degraded.

(Figure S1), where membranes are shown to be extant and intact throughout the volume of foam. SEM and light microscopy have been used to confirm the nature of membranes for devices prior to implantation.

Strut Edge Assumption

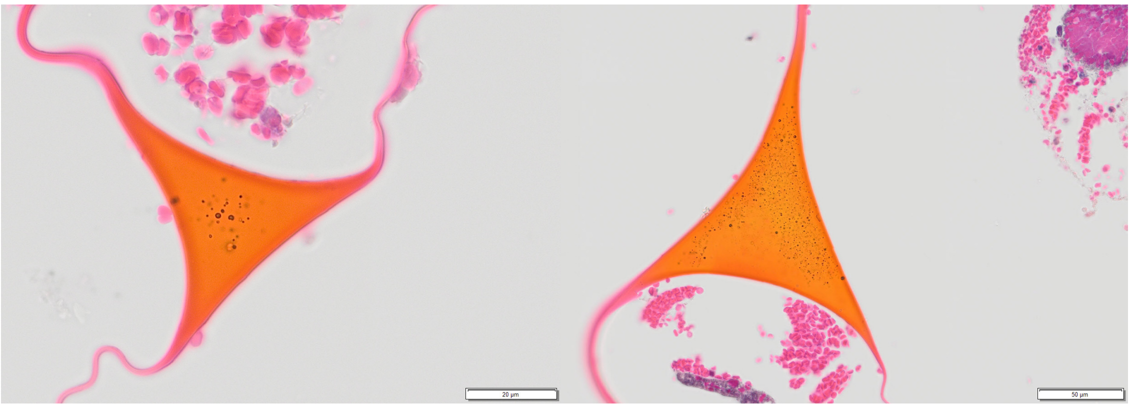

Figure S2: Struts from a non-degraded foam are shown in a sectional view. As seen for both struts, the sides have a smooth edge that curves dramatically when transitioning to a membranous form.

Struts prior to implantation are assumed to have smooth surfaces with few visible surface perturbations. Sectional images from 0-day foam explants show struts with smooth and well-defined edges (Figure S2).

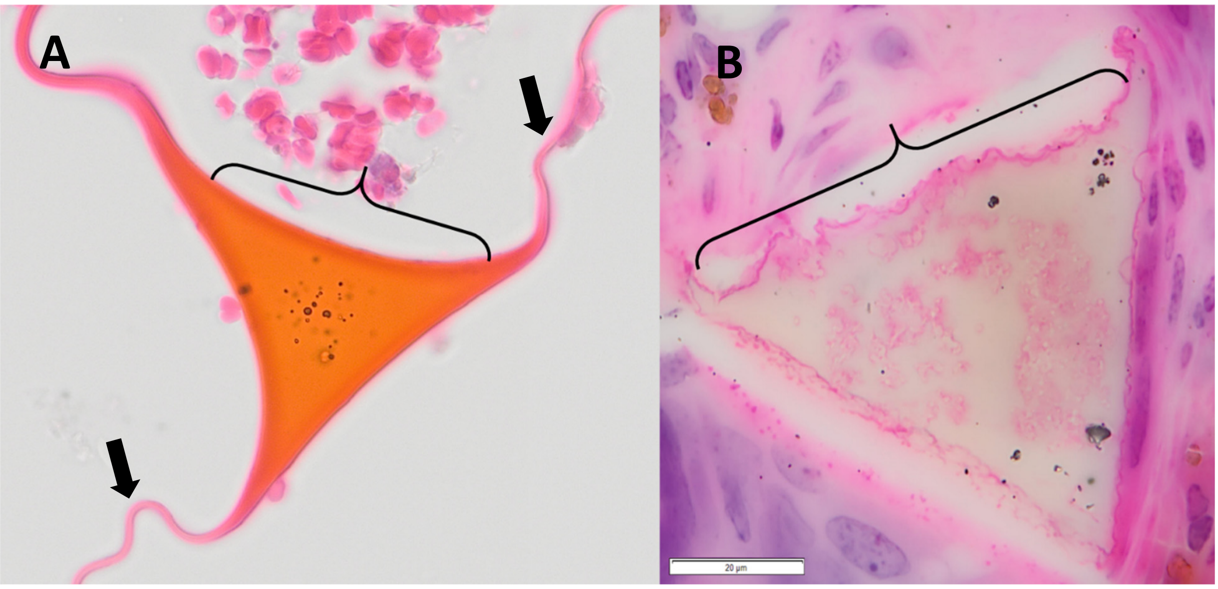

Figure S3: Strut edge smoothness can be observed at 0-days (A), where the edges seamlessly shift into membrane at the vertices. The membranous material (black arrows) presents with a looser geometry and conformation. As seen in (B), membranes have been separated from the vertices and degradation is present around the strut edges. The bracketed region shows an example of extreme variation in the edge due to degradation events.

The struts naturally thin at the vertices, where the strut transitions into membrane. This is supported by a visible difference in stain saturation and color, where struts present with a deep orange-yellow color and membranes appear as a pink-violet, with lower overall saturation. Images of struts where membranes have been separated from the vertices support this assumption, as membranes would be more susceptible to fracture. When struts are in states of degradation such as in Figure S3, evaluation of each degradation region relates back to the assumption of strut smoothness at implantation.
